# Epidemiology of drug–resistant tuberculosis in Hunan China over a 10–year period

**DOI:** 10.3389/fpubh.2026.1771140

**Published:** 2026-02-23

**Authors:** Yi Liu, Jue Wang, Xiaojie Wan, Wang Peng, Jingwei Guo, Xi Yang, Di Zhou, Wenbin Li, Jie Duan, Xuan Zeng, Hua Bai, Zhenhua Chen, Fangzhen Luo, Yunhong Tan

**Affiliations:** 1 Hunan Provincial Tuberculosis Prevention and Control Institute & Hunan Chest Hospital, Changsha, Hunan, China; 2 Guangzhou Huazhun Medical Laboratory Co., Ltd., Guangzhou, Guangdong, China; 3 Hunan Province Cooperative Innovation Center for Molecular Target New Drug Study, School of Pharmaceutical Science, Hengyang Medical School, University of South China, Hengyang, China

**Keywords:** China, drug–resistant tuberculosis, epidemiology, Hunan, *Mycobacterium tuberculosis*, retrospective study

## Abstract

**Background:**

Drug–resistant tuberculosis (DR–TB) remains a major public health challenge in China, yet long–term epidemiological data from key regions such as Hunan Province in South–Central China are still limited.

**Objective:**

This study aimed to characterize the epidemiological trends, spatial distribution, and risk factors of Single drug–resistant tuberculosis (SDR–TB), poly–drug–resistant tuberculosis (PDR–TB), multidrug–resistant tuberculosis (MDR–TB), rifampicin–resistant tuberculosis (RR–TB), and isoniazid–resistant tuberculosis (INH–R TB) in Hunan Province between 2014 and 2023, to inform region–specific control strategies.

**Methods:**

It was a retrospective analysis that was conducted on 6,597 laboratory–confirmed DR–TB cases. Data were obtained from the Provincial Tuberculosis Control Institute. All patients underwent phenotypic drug susceptibility testing. Independent risk factors for DR–TB subtypes were identified through multivariable logistic regression, with adjusted odds ratios (ORs) and corresponding 95% confidence intervals (CIs) calculated.

**Results:**

A total of 6,597 patients with DR–TB were included in this 10–year analysis. Among them, 74.97% were male and 64.44% were farmers. The highest case burden was observed in the 50–59 age group (24.78%). Spatially, cases clustered mainly in the Changsha (16.39%), Shaoyang (13.78%), and Loudi (9.05%). The most common resistance subtypes were INH–R TB (56.27%) and MDR–TB (52.37%). The distribution of all DR–TB subtypes varied significantly across age groups (*p* < 0.05), with peaks in middle–aged and older adults. Over time, the detection rate of MDR–TB was highest in 2018, while RR–TB remained the most frequently detected resistance type. Multivariable analysis identified significant regional and demographic disparities. The eastern region of Hunan was associated with an increased risk of SDR–TB (OR = 1.334) and PDR–TB (OR = 1.208), whereas the western region carried the highest risk for MDR–TB (OR = 1.734). Female patients consistently showed lower risks of MDR–TB (OR = 0.819) and RR–TB (OR = 0.784) compared with males.

**Conclusion:**

This study presents a 10–year epidemiological assessment of DR–TB in Hunan Province, China, covering 2014–2023. A disproportionately high burden was observed among middle–aged and older male farmers. The predominance of INH–R TB and MDR–TB, together with distinct regional and demographic risk profiles, underscores an urgent need to strengthen TB control measures. These results support the implementation of targeted interventions, including intensified screening in high–risk populations and in high–incidence areas, along with optimized treatment regimens, to curb the ongoing DR–TB epidemic in South–Central China.

## Introduction

Tuberculosis (TB) remains a leading cause of mortality from infectious diseases worldwide (1). The emergence and spread of Drug–resistant tuberculosis (DR–TB) have posed serious challenges to global TB control (2, 3). As one of the countries with the highest TB burden, China faces a particularly high prevalence of DR–TB, posing significant challenges to national TB control efforts (4). South–Central China, including Hunan Province, is characterized by a large population, substantial internal migration, and diverse socioeconomic conditions (5, 6). Although this region plays a critical role in national TB control, there is a lack of long–term systematic research on DR–TB in this area. A clear and comprehensive understanding of DR–TB trends and characteristics in this region is crucial for designing effective prevention and control strategies at regional and national levels.

The global incidence of DR–TB has increased, driven primarily by the widespread use of anti–TB drugs and inadequate adherence to treatment (7, 8). DR–TB can be classified into several resistance patterns (9). In this study, we focused on five major types: monoresistant tuberculosis (SDR–TB), poly–drug–resistant tuberculosis (PDR–TB), multidrug–resistant tuberculosis (MDR–TB), rifampicin–resistant tuberculosis (RR–TB), and isoniazid–resistant tuberculosis (INH–R TB). The management of DR–TB is prolonged and costly, with poorer treatment outcomes, placing substantial physical, psychological, and economic burdens on patients and creating major challenges for public health systems (10–12).

A clear understanding of the epidemiological characteristics, temporal trends in drug resistance, and related risk factors of DR–TB in South–Central China is vital for designing targeted interventions. To address this need, we conducted a 10–year retrospective analysis of DR–TB in the region to describe its epidemiology, assess temporal trends in drug resistance, and identify demographic and geographic risk factors.

## Materials and methods

### Data source

This study retrospectively analyzed data from patients diagnosed with TB at the Hunan Chest Hospital between January 1, 2014, and August 1, 2023. Located in South–Central China, the hospital is a provincial–level designated TB institute and clinical center. Demographic and clinical date–such as sex, age, occupation, place of residence, diagnostic results, and drug–susceptibility profiles–were extracted from the hospital’s information system. All personally identifiable data were anonymized prior to analysis to protect patient confidentiality.

### Inclusion and exclusion criteria

Eligible patients were residents of Hunan Province and had a laboratory–confirmed diagnosis of DR–TB, established through drug susceptibility testing (DST). Patients with a history of repeated hospital admissions, incomplete medical records, or residence outside Hunan Province were excluded. In total, 6,597 patients were included in the final analysis.

### Research methods

Phenotypic drug susceptibility testing for first–line anti–TB drugs–isoniazid (INH), rifampicin (RIF), ethambutol (EMB), and pyrazinamide (PZA)–was conducted using the absolute concentration method on solid media. Fresh colonies grown on Löwenstein–Jensen (L–J) medium were harvested, homogenized, and adjusted to a concentration of 1 mg/mL. The suspension was subsequently diluted 100–fold with sterile saline, and 0.1 mL of the diluted inoculum was inoculated onto solid medium slopes. After thorough mixing, the medium were incubated at 37 °C, and results were evaluated after 4 weeks. All procedures were performed in a Class II biosafety cabinet in accordance established with laboratory safety guidelines.

### Quality control

All laboratory staff underwent proficiency training provided by National Tuberculosis Reference Laboratory (NTRL). Each batch of culture medium underwent sterility testing and was verified with the H37Rv reference strain to ensure accuracy and reproducibility. All testing procedures were conducted in compliance with established quality–control standards.

### Statistical analysis

Data cleaning and statistical analysis were conducted in R (version 4.2.2) using RStudio. Figures were produced in R and Excel. Descriptive statistics summarized the distribution of DR–TB. Associations with age, sex, occupation, and region were examined using chi–square tests for categorical variables and Mann–Whitney U tests for continuous variables. Multivariable logistic regression was fitted including the following factors: year, region, age group, occupation and sex. Odds ratios (ORs) with 95% confidence intervals (CIs) and two–sided *P*–values were calculated; *p* < 0.05 was considered statistically significant.

## Results

### Epidemiology of DR–TB

**Figure 1:**
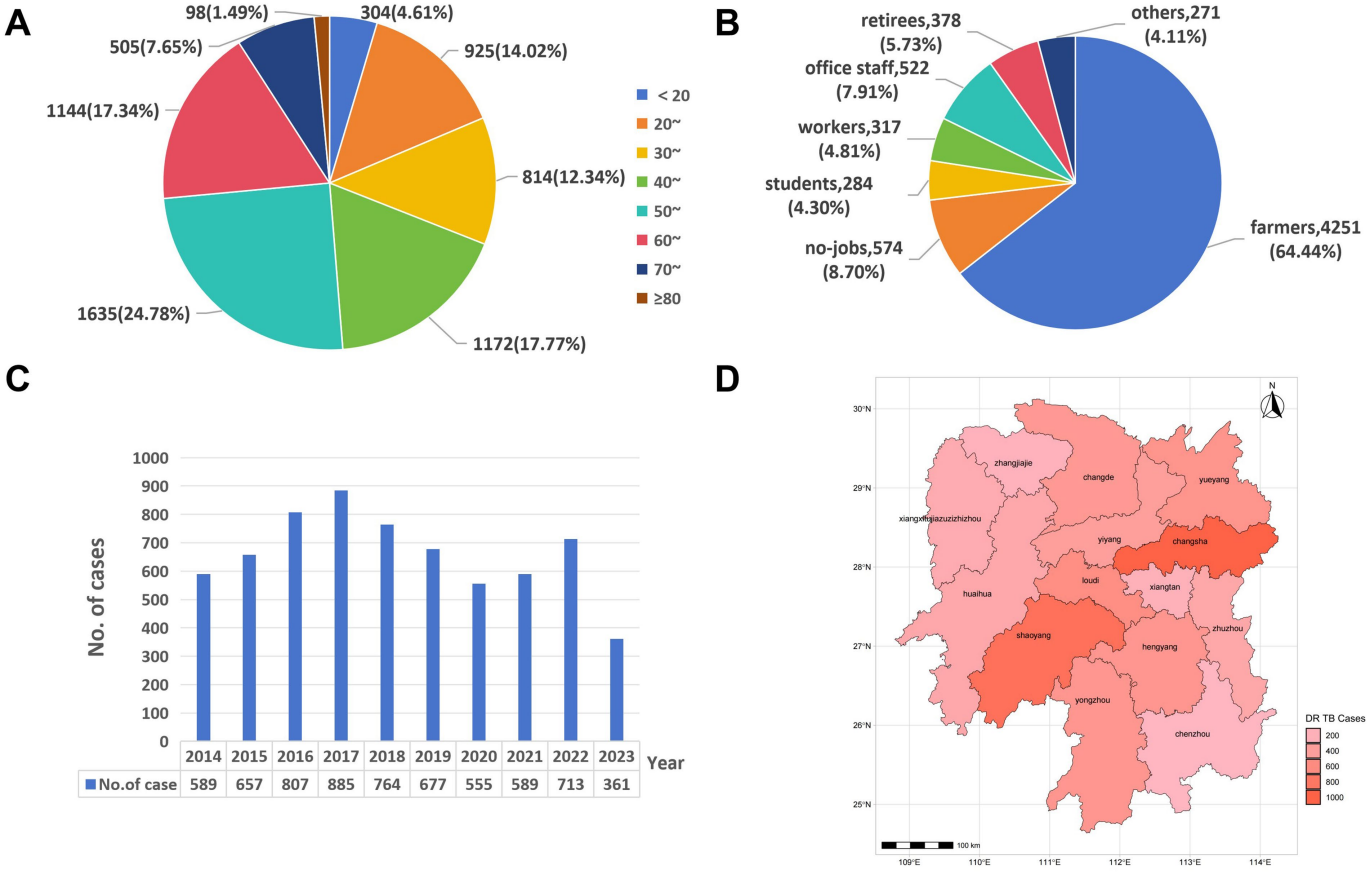
The distributions of DR–TB in Hunan, China, from 2014 to 2023. **(A)** Age distribution; **(B)** occupational distribution; **(C)** temporal distribution; and **(D)** spatial distribution.

This study included 6,597 patients with DR–TB diagnosed at Hunan Chest Hospital between 2014 and 2023. Among them, 4,946 were male and 1,651 were female, with a male–to–female ratio of 3.00:1. Ages ranged from 5 to 88 years, with a median of 50 years (interquartile range (IQR): 34–60). The 50–59 age group accounted for the highest proportion of cases (24.78%, 1,635/6,597), followed by the 60–69 age group (17.34%, 1,144/6,597; Figure 1A). Given the small number of cases aged < 10 years (*n* = 3), this group was merged with the ≤ 20 years category for subsequent analyses. By occupation, farmers accounted for the largest proportion of cases (64.44%, 4,251/6,597), followed by unemployed individuals (8.70%, 574/6,597) and public–sector employees (7.91%, 522/6,597) (Figure 1B). Temporal trend analysis (Figure 1C) showed that the number of DR–TB cases was relatively high between 2016–2018, peaking in 2017, and declined from 2019 to 2021. Regional analysis (Figure 1D) indicated that cases were concentrated in Changsha (16.39%, 1,081/6,597), Shaoyang (13.78%, 909/6,597), and Loudi (9.05%, 597/6,597), together accounting for 39.22% of all cases; fewer cases occurred in Xiangxi (0.26%, 17/6,597) and Zhangjiajie (1.00%, 66/6,597). Spatial distributions of DR–TB subtypes are shown in Appendix Table S1.

### Patterns of drug resistance subtypes

**Figure 2:**
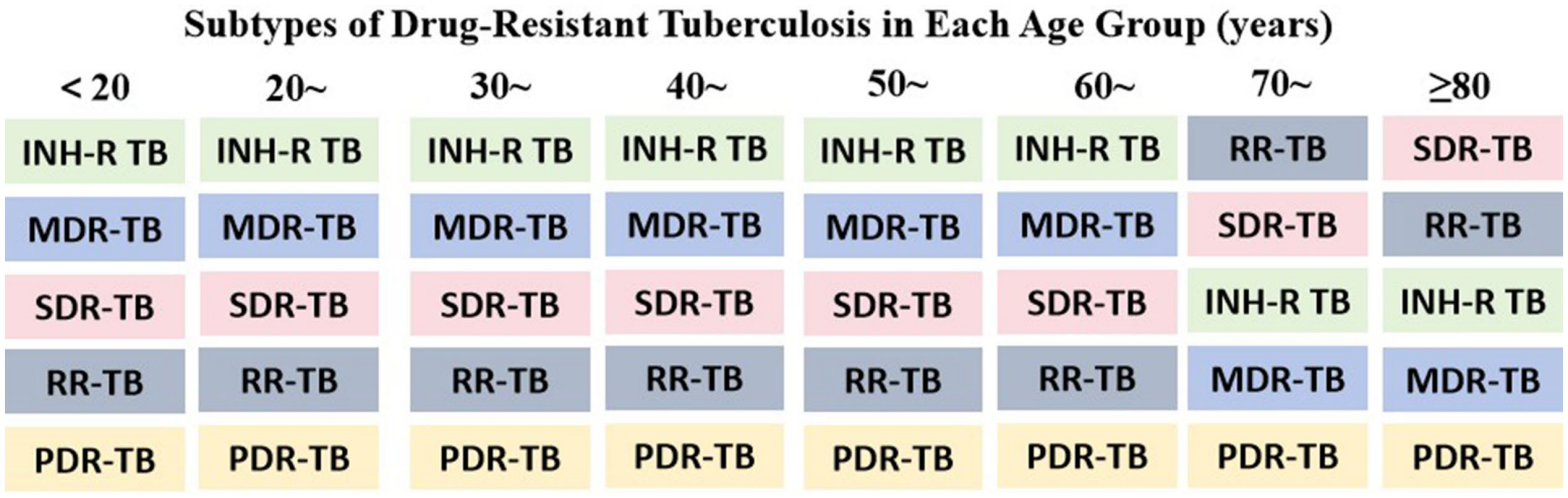
Subtypes of DR–TB in each age group (years). This figure illustrates the distribution of DR–TB subtypes by proportion across age groups (in descending order). The classification of DR–TB encompasses, but is not limited to, the subtypes displayed.

In this study, INH–R TB had the highest positivity rate (56.27%; 95% CI: 55.07–57.67), followed by MDR–TB (52.37%; 95% CI: 51.17–53.58). The rates for SDR–TB and RR–TB were similar, at 32.71% (95% CI: 31.58–33.84) and 31.47% (95% CI: 30.35–32.59), respectively. In contrast, PDR–TB had the lowest rate (13.75%; 95% CI: 12.92–14.58). Detailed results are shown in Table 1. The distribution of different DR–TB types across age groups was examined using the chi–square test, with results presented in Table 2. Among the 6,597 patients included, MDR–TB (3,455 cases) and INH–R TB (3,712 cases) were the most common types. Age distributions differed significantly across all resistance categories (*p* < 0.05). Overall, the number of DR–TB cases increased with age before declining, with a peak in the 50–59 age group. Specifically, INH–R TB and MDR–TB showed similar age–specific patterns, with the highest proportions in the 50–59 age group (INH–R TB: 961 cases, 25.9%; MDR–TB: 894 cases, 25.9%), followed by the 40–49 age group. In contrast, RR–TB, SDR–TB, and PDR–TB were also concentrated mainly in middle–aged and older adults, but their proportions within the ≥ 70 age group differed. Figure 2 illustrates the predominant subtypes of DR–TB across age groups.

**Table 1:** Prevalence and 95% CI of DR–TB subtypes.

Subtypes	Yes (*n*, %)	No (*n*, %)	Proportion (95% CI)
SDR–TB	2,158 (32.71)	4,439 (67.29)	32.71 (31.58–33.84)
PDR–TB	907 (13.75)	5,690 (86.25)	13.75 (12.92–14.58)
MDR–TB	3,455 (52.37)	3,142 (47.63)	52.37 (51.17–53.58)
RR–TB	2076 (31.47)	4,521 (68.53)	31.47 (30.35–32.59)
INH–R TB	3,712 (56.27)	2,885 (43.73)	56.27 (55.07–57.67)

TB, tuberculosis; DR–TB, drug–resistant tuberculosis; SDR–TB, single drug–resistant tuberculosis; PDR–TB, poly–drug–resistant tuberculosis; MDR–TB, multidrug–resistant tuberculosis; RR–TB, rifampicin–resistant tuberculosis; INH–R TB, isoniazid–resistant tuberculosis. The classification of DR–TB includes, but is not limited to, the subtypes listed.

**Table 2:** Distribution of DR–TB subtypes across different age groups.

Subtypes	No. of cases in each age group (years)								Total	*χ^2^*	*p*
	<20	20~	30~	40~	50~	60~	70~	≥80			
SDR–TB	111	335	228	310	504	376	244	50	2,158	109.656	0.000
PDR–TB	50	127	100	139	220	164	84	23	907	18.667	0.009
MDR–TB	136	456	476	711	894	590	168	24	3,455	163.308	0.000
RR–TB	97	291	219	304	498	381	277	40	2076	105.798	0.000
INH–R TB	144	488	505	758	961	641	188	27	3,712	170.558	0.000

The classification of DR–TB includes, but is not limited to, the subtypes presented. Analysis of variance was applied for intergroup comparisons, *p* < 0.05.

### Temporal profiling of DR–TB

**Figure 3:**
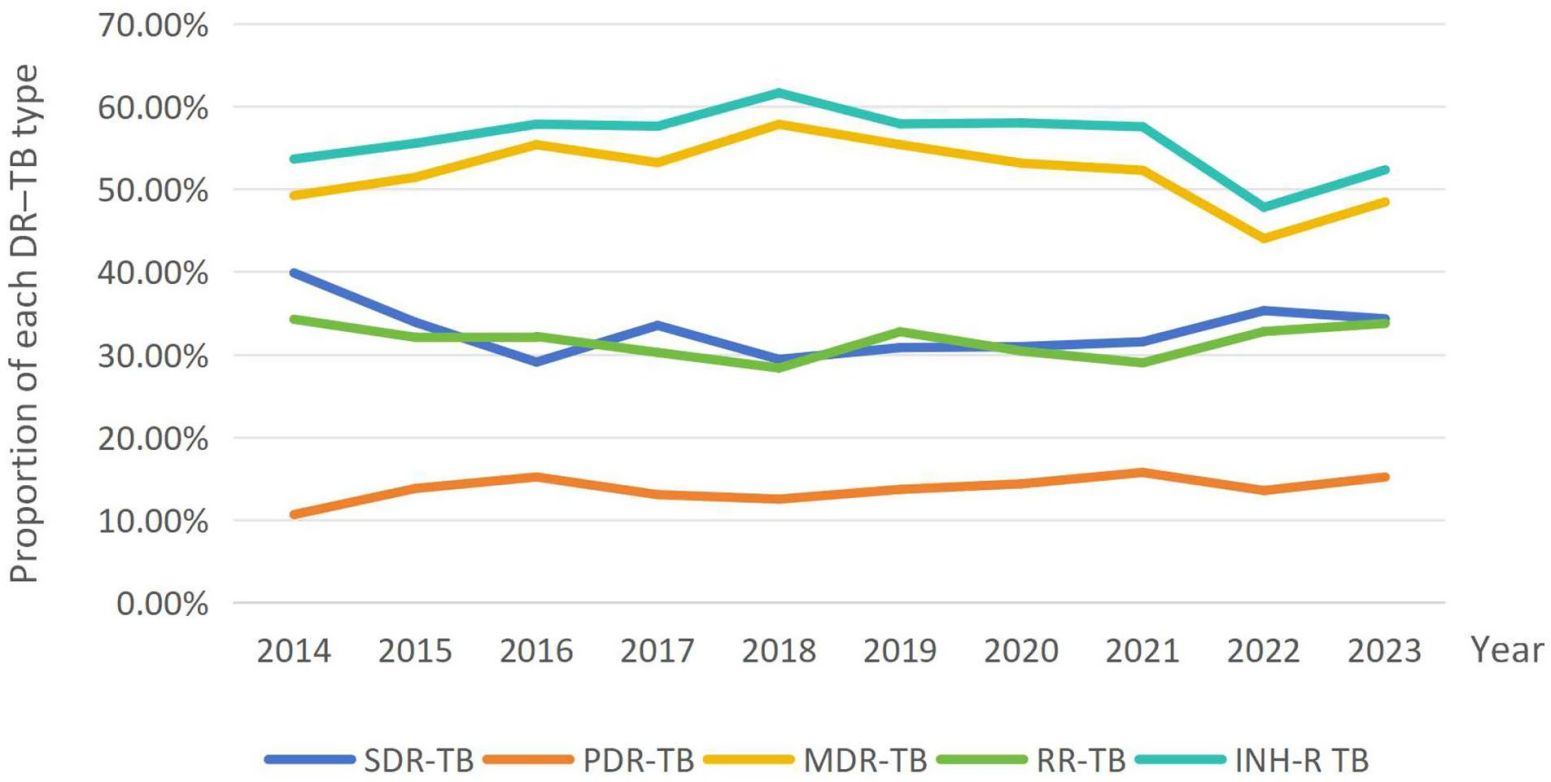
Temporal trends in detection rates of different DR–TB types, 2014–2023. The *Y*–axis represents the proportion of each DR–TB type, calculated as its case count divided by the total number of DR–TB cases for that year.

From 2014 to 2023, detection rates of the different DR–TB types showed distinct patterns (Figure 3). RR–TB consistently had the highest rate and displayed a temporal trend similar to MDR–TB. The MDR–TB rate peaked in 2018, then declined with fluctuations. SDR–TB and INH–R TB remained relatively stable, generally ranging from 30 to 40%, with parallel trends and only minor variations. PDR–TB had the lowest rate throughout the study period. Chi–square tests indicated that, except for RR–TB, distributions of the other subtypes varied significantly by year (SDR–TB, PDR–TB, MDR–TB, and INH–R TB: *p* < 0.05; *χ*^2^ = 27.797, 10.322, 39.609, and 36.990, respectively). Detailed results are provided in Appendix Table S1.

### Differences by region, sex, and occupation

Based on geographic location, the 14 prefecture–level divisions of Hunan Province were grouped into five regions: eastern (Changsha, Xiangtan, Zhuzhou), southern (Hengyang, Yongzhou, Chenzhou), western (Zhangjiajie, Xiangxi, Huaihua), northern (Yueyang, Changde), and central (Loudi, Yiyang, Shaoyang). Regional analysis showed significant differences in the distribution of SDR–TB, PDR–TB, MDR–TB, RR–TB, and INH–R TB across these regions (*χ*^2^ = 134.501, 16.524, 197.853, 123.445, and 165.209, respectively; *p* < 0.05). Gender–based analysis revealed that males constituted a significantly higher proportion of cases than females for SDR–TB, MDR–TB, RR–TB, and INH–R TB (*χ*^2^ = 4.350, 163.308, 105.798, and 170.558, respectively; *p* < 0.05). Regarding occupational distribution, farmers represented the largest subgroup within the study population. Chi–square tests indicated significant variation in the distribution of SDR–TB, MDR–TB, RR–TB, and INH–R TB across occupational categories (*χ*^2^ = 39.165, 58.294, 34.275, and 64.518, respectively; *p* < 0.05). Detailed results are shown in Appendix Table S1 and Supplementary Figure S1.

### Multivariable analysis of risk factors

**Figure 4:**
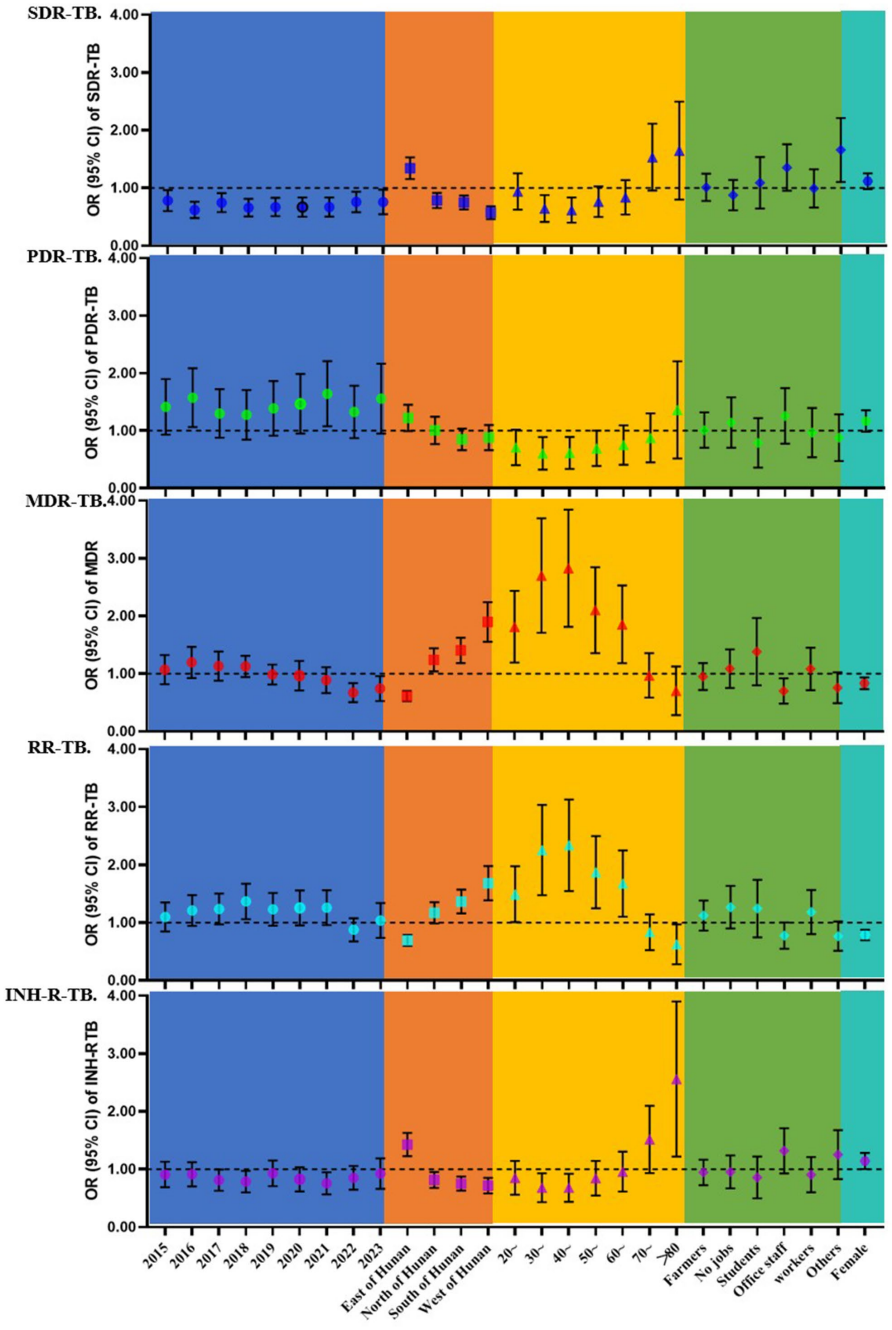
Forest plot of the multivariable analysis. Forest plot of multivariable analyses showing the associations between various risk factors and different DR–TB subtypes.

The results of the multivariate logistic regression analysis are summarized in Figure 4. For SDR–TB, the incidence risk generally decreased from 2015 to 2023 compared with 2014. Geographically, the eastern region had a significantly higher risk (OR = 1.334), whereas the northern, southern, and western regions had lower risks. For PDR–TB, the risk was significantly higher in 2016 (OR = 1.515) and 2021 (OR = 1.574) compared with 2014, and the eastern region also showed increased risk (OR = 1.208). For MDR–TB, the risk was significantly elevated in 2016, 2018, and 2019 compared with 2014. Spatially, the eastern region had lower risk (OR = 0.658), whereas the northern (OR = 1.219), southern (OR = 1.388), and western (OR = 1.734) regions had higher risks. Individuals aged 20–69 years were at significantly higher risk than those under 20 years, and females had a lower risk than males (OR = 0.819). For RR–TB, the risk increased significantly in 2018 (OR = 1.344). The eastern region had lower risk, while the southern and western regions had higher risks. The 20–69 age group had elevated risk, and females had a lower risk than males (OR = 0.784). For INH-R TB, the risk decreased in 2018 and 2021. The eastern region showed higher risk, whereas other regions had lower risks. Females were at significantly higher risk than males (OR = 1.137). Detailed results are provided in Appendix Table S2.

## Discussion

This 10–year retrospective study examines the DR–TB epidemic in Hunan Province, South–Central China. The analysis identifies distinct temporal, geographic, and demographic patterns, providing evidence to inform targeted public health strategies.

The predominance of RR–TB and INH–R TB among resistance types is noteworthy. Both showed temporal trends similar to MDR–TB, with all three peaking in 2018. This pattern may be attributed to the widespread use of RIF and INH as first–line anti–TB drugs. Inconsistent treatment adherence and non–standardized medication practices can create selective pressure, promoting the emergence and transmission of resistant strains. This phenomenon aligns with findings from other high–TB–burden regions (13, 14). The subsequent rebound in resistance rates observed in 2023 may reflect disruptions in TB control services during the COVID–19 pandemic, such as resource reallocation and interruptions in patient follow–up (15–17), underscoring the necessity for sustained vigilance in the post–pandemic era. It should be noted, however, that data collection for 2023 was incomplete due to a hospital information system upgrade concluding in August, which may have influenced the annual estimates.

Geographically, we observed clear spatial disparities in the distribution of DR–TB. The western and southern regions of Hunan had the highest burden of MDR–TB, showing an inverse association with socioeconomic development, consistent with prior studies (18–20). This pattern may reflect limited healthcare access and suboptimal treatment adherence in the mountainous west. In the south, a major source of out–migrant labor, treatment interruption among mobile populations could facilitate transmission of resistant strains (21, 22). By contrast, the more developed eastern region showed lower odds of MDR–TB (OR = 0.658), likely due to stronger TB control infrastructure, including broader DOTS coverage and wider availability of rapid molecular DST (23).

Across most DR–TB subtypes, male patients constituted a significantly higher proportion of cases (73–77%), exceeding the corresponding figures reported in countries such as Mexico and Japan (24, 25). This gender disparity may be attributable to factors including a higher prevalence of smoking among men–which can impair pulmonary immunity–greater occupational exposure to silica dust and other hazardous agents (e.g., in mining or construction), and a tendency to delay seeking medical care (26–28). Individuals aged 30–59 years exhibited a higher incidence of MDR–TB and RR–TB, indicating increased vulnerability potentially linked to high social mobility, greater work–related stress, and heavier comorbidity burdens. Although healthcare resources for this age group are generally sufficient, competing job demands and delayed healthcare–seeking may limit their timely access to TB services.

Farmers accounted for the majority of DR–TB cases (64.44%), highlighting substantial weaknesses in rural TB control. This vulnerability may be linked to insufficient health insurance, low health literacy, physically demanding work, and poor nutrition among agricultural workers, factors that can elevate the risk of TB infection and drug resistance (29–31). The unemployed comprised 8.70% of cases; we speculate that many lost their work capacity due to TB, with unemployment further aggravating economic and social marginalization, thereby perpetuating a vicious cycle of poverty and disease. This pattern underscores the profound impact of DR–TB on patients’ livelihoods and social functioning.

Multivariable logistic regression confirmed an elevated risk of MDR–TB in the western region (OR = 1.734) and among adults aged 30–49 years (OR > 2.0). Public sector employees had a significantly lower risk compared with farmers (OR = 0.710), suggesting that occupational disparities in healthcare access are a potential target for intervention (32). The modestly increased risk of INH–R TB among females (OR = 1.137) warrants further investigation into potential sex–based differences in drug metabolism (e.g., N–acetyltransferase 2 activity) and caregiving–related exposure (33, 34).

Despite insights from this large, decade–long analysis of the DR–TB epidemic in Hunan Province, several limitations should be considered. First, the single–center, retrospective design may limit generalizability and preclude causal inference. Second, the absence of genotyping data limits analysis of transmission dynamics and molecular clustering. Third, missing data on key confounders–including HIV or diabetes comorbidity, behavioral factors (e.g., smoking and alcohol use), and detailed treatment history–may bias risk–factor analyses. Finally, because some second–line and newer antituberculosis drugs were introduced unevenly during the study period, drug–resistant tuberculosis (XDR–TB) and pre–extensively drug–resistant tuberculosis (pre–XDR–TB) were not included in the epidemiologic analyses.

Future multicenter, prospective studies that incorporate whole–genome sequencing (WGS) and qualitative assessments of treatment adherence are needed to address these limitations and clarify the epidemiology of DR–TB.

## Conclusion

The DR-TB epidemic in Hunan Province shows marked spatiotemporal heterogeneity and distinctive demographic clustering. To address these challenges, precision public health interventions are essential. Key priorities include (1) expanding the use of rapid molecular diagnostics and DST in high–burden western and southern regions; (2) implementing flexible, community–based directly observed therapy programs tailored to farmers and migrant populations; and (3) enhancing medical security and health education for high–risk occupational groups through coordinated cross–sectoral action. These findings provide a robust evidence base for developing region-specific DR–TB control guidelines in China and comparable settings.

## Data Availability

The raw data supporting the conclusions of this article will be made available by the authors, without undue reservation.
