# Supplementary figures and images for: Epidemiology of drug–resistant tuberculosis in Hunan China over a 10–year period
Source: Front Public Health. 2026 Feb 23;14:1771140. doi: 10.3389/fpubh.2026.1771140 (PMC12968256; doi:10.3389/fpubh.2026.1771140)

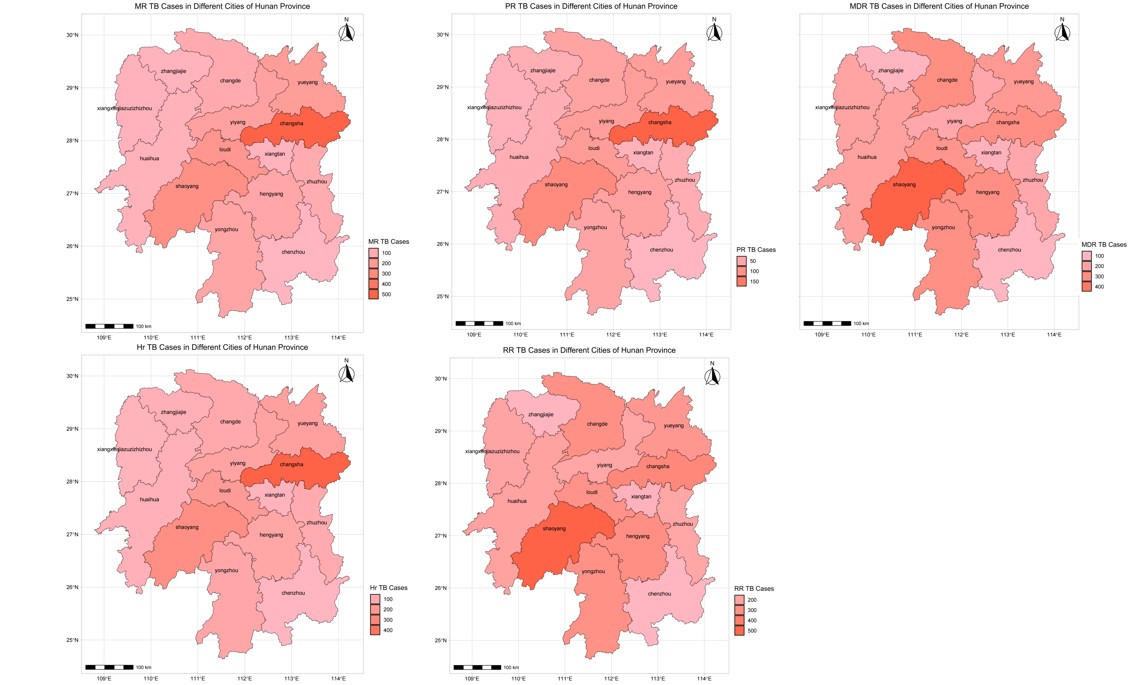

Supplement: Supplementary file 1 [file Image_1.jpeg]
