# Supplementary material for: Epidemiology of drug–resistant tuberculosis in Hunan China over a 10–year period
Source: Front Public Health. 2026 Feb 23;14:1771140. doi: 10.3389/fpubh.2026.1771140 (PMC12968256; doi:10.3389/fpubh.2026.1771140)
Supplement: Supplementary file 3 [file Table_2.docx]

Appendix 1-Table S2 Associated factors with DR-TB using multivariable logistic regression.

| Characteristics | SDR-TB/TB(n,%) | P | OR (95% Cl) | PDR-TB/TB(n,%) | P | OR (95% Cl) | MDR/TB(n,%) | P | OR (95% Cl) | INH-R TB/TB(n,%) | P | OR (95% Cl) | RR-TB/TB(n,%) | P | OR (95% Cl) |
| --- | --- | --- | --- | --- | --- | --- | --- | --- | --- | --- | --- | --- | --- | --- | --- |
| Year of onset | | | | | | | | | | | | | | | |
| 2014 | 235, 39.90% | Reference |  | 63 | Reference |  | 268, 47.69% | Reference |  | 202, 34.30% | Reference |  | 316, 53.65% | Reference |  |
| 2015 | 223, 33.94% | 0.028* | 0.767(0.605-0.971) | 91, 13.85% | 0.083 | 1.358(0.963-1.924) | 311, 49.76% | 0.686 | 1.050 (0.828-1.332) | 211, 32.12% | 0.377 | 0.879 (0.704-1.142) | 365, 55.56% | 0.515 | 1.080 (0.857-1.360) |
| 2016 | 235, 29.12% | 0.000* | 0.609(0.483-0.767) | 123, 15.24% | 0.013* | 1.515(1.095-2.114) | 420, 54.26% | 0.167 | 1.175 (0.935-1.476) | 260, 32.22% | 0.349 | 0.895 (0.711-1.129) | 467, 57.87% | 0.119 | 1.193 (0.956-1.488) |
| 2017 | 297, 33.56% | 0.006* | 0.734(0.588-0.916) | 116, 13.11% | 0.182 | 1.251(0.904-1.746) | 452, 52.74% | 0.349 | 1.113 (0.890-1.392) | 268, 30.28% | 0.052 | 0.798 (0.635-1.002) | 510, 57.63% | 0.074 | 1.218 (0.981-1.513) |
| 2018 | 225, 29.45% | 0.000* | 0.647(0.513-0.817) | 96, 12.57% | 0.244 | 1.225(0.873-1.730) | 423, 57.63% | 0.054 | 1.257 (0.996-1.586) | 217, 28.40% | 0.032* | 0.771 (0.608-0.978) | 471, 61.65% | 0.010* | 1.344 (1.073-1.684) |
| 2019 | 209, 30.87% | 0.001* | 0.658(0.518-0.835) | 93, 13.74% | 0.103 | 1.333(0.946-1.888) | 328, 56.94% | 0.424 | 1.107 (0.863-1.1.422) | 222, 32.79% | 0.449 | 0.911 (0.717-1.159) | 392, 57.90% | 0.107 | 1.209 (0.960-1.523) |
| 2020 | 172, 30.99% | 0.001* | 0.655(0.509-0.842) | 80, 14.41% | 0.061 | 1.406(0.985-2.015) | 257, 53.54% | 0.645 | 0.940 (0.723-1.233) | 169, 30.45% | 0.103 | 0.809 (0.626-1.043) | 322, 58.02% | 0.093 | 1.231 (0.966-1.569) |
| 2021 | 186, 31.58% | 0.001* | 0.655(0.511-0.840) | 93, 15.79% | 0.011* | 1.574(1.113-2.239) | 281, 50.91% | 0.283 | 0.870 (0.674-1.122) | 171, 29.03% | 0.020* | 0.740 (0.573-0.954) | 339, 57.56% | 0.084 | 1.236 (0.972-1.572) |
| 2022 | 252, 35.34% | 0.014* | 0.744(0.587-0.942) | 97, 13.60% | 0.172 | 1.273(0.902-1.808) | 297, 44.07% | 0.001* | 0.659 (0.514.0.845) | 234, 32.82% | 0.142 | 0.834 (0.655-1.063) | 341, 47.83% | 0.207 | 0.862 (0.684-1.085) |
| 2023 | 124, 34.35% | 0.035* | 0.738(0.555-0.978) | 55, 15.24% | 0.052 | 1.480(0.994-2.198) | 165, 47.69% | 0.029* | 0.722 (0.538-0.967) | 122, 33.80% | 0.474 | 0.900 (0.675-1.199) | 189, 52.35% | 0.993 | 0.999 (0.759-1.359) |
| Region | | | | | | | | | | | | | | | |
| Center of Hunan (Loudi, Yiyang, Shaoyang) | 671, 34.69% | Reference |  | 266, 13.75% | Reference |  | 922 | Reference |  | 624, 32.26% | Reference |  | 1060, 54.81% | Reference |  |
| East of Hunan (Changsha, Xiangtan, and  Zhuzhou) | 690, 42.15% | 0.000* | 1.334(1.160-1.535) | 268, 16.37% | 0.049* | 1.208(1.000-1.459) | 575 | ＜0.001 | 0.608 (0.525-0.703) | 676, 41.30% | ＜0.001* | 1.417 (1.231-1.633) | 733, 44.78% | ＜0.001* | 0.690 (0.601-0.791) |
| North of Hunan (Yueyang, Changde) | 295, 28.84% | 0.003* | 0.776(0.656-0.917) | 138, 13.49% | 1.000 | 1.000(0.770-1.247) | 537 | 0.012 | 1.230 (1.046-1.446) | 279, 27.27% | 0.013* | 0.807 (0.681-0.956) | 608, 59.43% | 0.061 | 1.162 (0.993-1.359) |
| South of Hunan (Hengyang, Yongzhou, and  Chenzhou) | 322, 26.90% | 0.000* | 0.742(0.631-0.871) | 138, 11.53% | 0.112 | 0.836(0.668-1.041) | 671 | ＜0.001 | 1.392 (1.191-1.627) | 300, 25.06% | ＜0.001* | 0.745 (0.632-0.877) | 765, 63.91% | ＜0.001* | 1.356 (1.166-1.578) |
| West of Hunan (Zhangjiajie, xiangxi, and  Huaihua) | 180, 22.33% | 0.000* | 0.566(0.466-0.686) | 97, 12.03% | 0.255 | 0.865(0.671-1.107) | 496 | ＜0.001 | 1.875 (1.565-2.251) | 197, 24.44% | ＜0.001* | 0.710 (0.587-0.856) | 524, 65.01% | ＜0.001* | 1.664 (1.396-1.987) |
| Age groups(years) | | | | | | | | | | | | | | | |
| ＜20 | 111, 36.51% | Reference |  | 50, 16.45% | Reference |  | 118 | Reference |  | 97, 31.91% | Reference |  | 144, 47.37% | Reference |  |
| 20~ | 335, 36.22% | 0.554 | 0.903(0.644-1.269) | 127, 13.73% | 0.599 | 0.662(0.426-1.038) | 430 | 0.002 | 1.741 (1.233-2.470) | 291, 31.46% | 0.258 | 0.818 (0.579-1.160) | 488, 52.76% | 0.029* | 1.440 (1.039-1.999) |
| 30~ | 228, 28.01% | 0.009* | 0.616(0.428-0.890) | 100, 12.29% | 0.069 | 0.560(0.351-0.908) | 432 | ＜0.001 | 2.577 (1.779-3.750) | 219, 26.90% | 0.023* | 0.648 (0.447-0.943) | 505, 62.04% | ＜0.001* | 2.165 (1.525-3.079) |
| 40~ | 310, 26.45% | 0.004* | 0.590(0.413-0.845) | 139, 11.86% | 0.017* | 0.569(0.361-0.911) | 673 | ＜0.001 | 2.704 (1.883-3.901) | 304, 25.94% | 0.020* | 0.649 (0.452-0.936) | 758, 64.68% | ＜0.001* | 2.249 (1.597-3.172) |
| 50~ | 504, 30.83% | 0.081 | 0.731(0.516-1.041) | 220, 13.46% | 0.017* | 0.644(0.413-1.024) | 824 | ＜0.001 | 2.010 (1.406-2.888) | 498, 30.46% | 0.243 | 0.808 (0.566-1.159) | 961, 58.78% | 0.001* | 1.801 (1.285-2.528) |
| 60~ | 376, 32.87% | 0.225 | 0.800(0.559-1.150) | 164, 14.34% | 0.058 | 0.695(0.439-1.117) | 549 | 0.002 | 1.771 (1.227-2.567) | 381, 33,30% | 0.634 | 0.915 (0.635-1.323) | 641, 56,03% | 0.007 * | 1.610 (1.139-2.280) |
| 70~ | 244, 48.32% | 0.053 | 1.460(0.996-2.147) | 84, 16.63% | 0.126 | 0.804(0.491-1.333) | 155 | 0.681 | 0.919 (0.614-1.378) | 228, 45.15% | 0.067 | 1.439 (0.976-2.132) | 188, 37.23% | 0.238 | 0.797 (0.547-1.162) |
| ＞80 | 50, 51.02% | 0.136 | 1.500(0.881-2.558) | 23, 23.47% | 0.391 | 1.191(0.616-2.278) | 21 | 0.141 | 0.624 (0.328-1.156) | 58, 59.18% | 0.002* | 2.320 (1.354-4.005) | 27, 27.55% | 0.044* | 0.562 (0.317-0.997) |
| Career | | | | | | | | | | | | | | | |
| Retirees | 151, 39.95% | Reference |  | 63, 16.67% | Reference |  | 148 | reference |  | 157, 41.53% | reference |  | 169, 44.71% | reference |  |
| Farmers | 1335, 31.40% | 0.944 | 0.992(0.785-1.256) | 563, 13.24% | 0.876 | 0.976(0.723-1.334) | 2150 | 0.572 | 0.931 (0.726-1.194) | 1287, 30.28% | 0.528 | 0.928 (0.735-1.173) | 2490, 58.57% | 0.407 | 1.103(0.875-1.392) |
| No jobs | 160, 27.87% | 0.292 | 0.849(0.627-1.151) | 85, 14.81% | 0.684 | 1.084(0.735-1.605) | 293 | 0.750 | 1.052 (0.771-1.436) | 167, 29.09% | 0.610 | 0.925 (0.684-1.250) | 346,60.28% | 0.161 | 1.233(0.920-1.653) |
| Students | 104, 36.62% | 0.895 | 1.029(0.676-1.564) | 39, 13.73% | 0.239 | 0.711(0.401-1.251) | 124 | 0.238 | 1.297 (0.843-2.001) | 85, 29.93% | 0.333 | 0.808 (0.525-1.241) | 141, 49.65% | 0.434 | 1.177(0.783-1.772) |
| Office staff | 199, 38.12% | 0.074* | 1.314(0.974-1.775) | 85, 16.28% | 0.373 | 1.194(0.809-1.770) | 208 | 0.015* | 0.677 (0.494-0.929) | 194, 37.16% | 0.106 | 1.280 (0.949-1.728) | 248, 47.51% | 0.061 | 0.754(0.561-1.013) |
| workers | 95, 29.97% | 0.786 | 0.954(0.679-1.338) | 39, 12.30% | 0.671 | 0.906(0.572-1.423) | 168 | 0.819 | 1.041 (0.738-1.471) | 89, 28.08% | 0.432 | 0.872 (0.619-1.225) | 189, 59.62% | 0.425 | 1.142(0.825-1.584) |
| Others | 114, 42.07% | 0.007 | 1.597(1.137-2.241) | 33, 12.18% | 0.414 | 0.820(0.506-1.311) | 111 | 0.078 | 0.725 (0.506-1.037) | 97, 35.79% | 0.286 | 1.205 (0.855-1.697) | 129, 47.60% | 0.077 | 0.738(0.528-1.033) |
| Sex | | | | | | | | | | | | | | | |
| male | 1583, 32.01% | Reference |  | 657, 13.28% | Reference |  | 2459 | reference |  | 1522, 30.77% | reference |  | 2459, 53.18% | reference |  |
| Female | 575, 34.83% | 0.088 | 1.112(0.984-1.256) | 250, 15.14% | 0.072 | 1.159(0.986-1.360) | 743 | 0.003* | 0.829 (0.734-0.937) | 554, 33.56% | 0.041* | 1.137 (1.005-1.285) | 743, 47.75% | ＜0.001* | 0.784 (0.698-0.881) |

Note: **P* < 0.05, The DR-TB classification is not limited to the specific subtypes listed in this table.
